# Sealing porous anodic layers on AA2024-T3 with a low viscosity benzoxazine resin for corrosion protection in aeronautical applications

**DOI:** 10.1039/c9ra01970g

**Published:** 2019-06-06

**Authors:** Alexis Renaud, Yoann Paint, Alex Lanzutti, Leïla Bonnaud, Lorenzo Fedrizzi, Philippe Dubois, Marc Poorteman, Marie-Georges Olivier

**Affiliations:** Department of Materials Science, Materials Engineering Research Center (CRIM), University of Mons Place du Parc 20 B-7000 Mons Belgium marc.poorteman@umons.ac.be; Materia Nova Asbl Avenue Copernic 1 B-7000 Mons Belgium; Polytechnic Department of Engineering and Architecture, University of Udine Via del Cotonificio 108 33100 Udine Italy; Laboratory of Polymeric and Composite Materials, Center of Innovation and Research in Materials and Polymers (CIRMAP), University of Mons Place du Parc 20 B-7000 Mons Belgium

## Abstract

In this paper, a 4-ethylphenol-*para*-phenylenediamine (4EP-*p*PDA) benzoxazine has been applied and cured on previously anodized AA2024-T3 substrates. The porous surface oxide layers obtained from sulfo-tartaric anodizing appeared to be highly impregnated by the benzoxazine resin, sealing the anodic films. Through rheological, morphological and chemical characterization, the curing process has been identified to be the key step for the impregnation to occur, related to the low viscosity of the 4EP-*p*PDA benzoxazine attained during thermal curing. Moreover, the typical surface porosity of the anodic layer reappeared after curing, offering a good anchoring to possible top coats. Finally, high and enduring barrier properties of this hybrid organic–inorganic layer have been highlighted through Electrochemical Impedance Spectroscopy (EIS) and correlated with recent results obtained by Molecular Dynamics Simulations (MDS). These barrier properties appeared to be strongly influenced by the curing process parameters, as has been assessed using alternative curing cycles limiting their duration and lowering the curing temperature. Consequently, adapting the curing process enables the optimization of the barrier properties of the system while respecting the dependence of the mechanical properties of the AA2024-T3 substrate on thermal treatment at high temperatures.

## Abstract 2

In this paper, a 4-ethylphenol-*para*-phenylenediamine (4EP-*p*PDA) benzoxazine has been applied and cured on previously anodized AA2024-T3 substrates.

## Graphical abstract

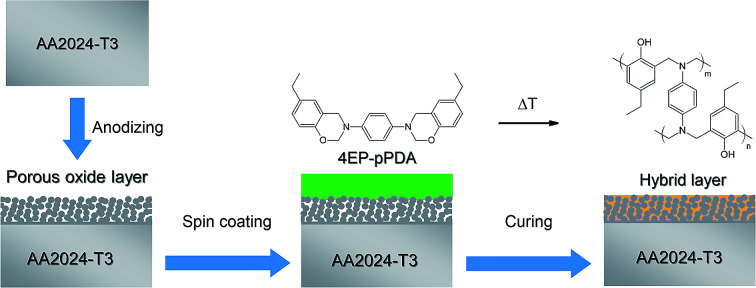

## 1 Introduction

Corrosion protection of aluminum is of major concern in the aircraft industry. Indeed, the good mechanical properties of several aluminum alloys achieved through control of their composition and microstructure, induce an increased sensitivity to corrosive environments.^1–3^ Until now, a very efficient protection system, consisting of a porous oxide layer obtained from chromic acid anodizing (CAA) and coated with an epoxy-based primer filled with corrosion inhibition pigments,^4^ has been employed. The elaboration of these systems involves hexavalent chromium species, providing efficient and durable corrosion protection in case of surface defect/damage.^5^ However, these compounds are toxic to human health and to the environment and, therefore, alternative protective systems must be developed.

Concerning anodization, different kinds of acid electrolyte baths have been investigated to replace chromic acid for anodizing of aluminum substrates,^4^ using either sulfuric acid (SAA),^6^ sulfo-tartaric acid (TSA)^7^ or sulfo-boric acid (BSA).^8^ By combining the strong sulfuric acid with the weak tartaric acid, the oxide dissolution is limited, and, therefore, a better control of the porosity is achieved, using low cost chemicals.^9^ The oxide layers obtained in sulfo-tartaric baths show a better corrosion resistance compared to those obtained from classical sulfuric acid baths^10^ and are currently qualified and implemented in the aerospace industry as a promising alternative for chromic anodizing. Sulfo-tartaric anodizing is usually performed at 37 °C under an applied potential difference of 14 V for about 20 or 30 minutes. The obtained oxide layers have a thickness ranging from 2 to 7 μm.

Concerning the organic primer part, polymeric materials other than classical epoxy resins can be considered as potential alternatives. In particular, benzoxazine resins are progressively getting the interest of the scientific community due to their good thermal and mechanical properties and high chemical resistances.^11–13^ Their potential to be used as protective coating materials has already been demonstrated in several recent studies, including also composite systems.^14–19^ More specifically, a phenol-*para*-phenylenediamine (P-*p*PDA) benzoxazine applied on either AA1050 or AA2024-T3 substrates has shown promising corrosion barrier properties, appearing to be strongly related to the curing parameters.^20^ Coating of a P-*p*PDA resin on top of anodic layers elaborated by sulfo-tartaric anodizing of aluminum AA2024-T3 substrates^20^ resulted in a good resin anchoring due to the favorable interaction between the resin and the porous anodic layer, providing an excellent durability of the barrier properties over immersion time in a saline solution, preventing or limiting delamination processes as observed on clear benzoxazine coatings.^17,20^ However, no proper impregnation of the porous oxide structure by the resin has been observed. The high viscosity of the P-*p*PDA precursor, even at high temperature, appears to be a limiting factor for the impregnation to occur.

**Fig. 1:**
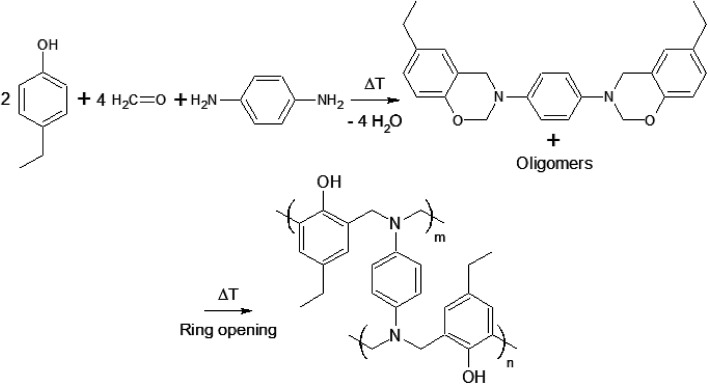
4EP-*p*PDA precursor synthesis and cross-linking.

In a recent paper, crosslinking during thermal curing of several benzoxazine thermosets has been modeled through Molecular Dynamics Simulations (MDS) by some of the co-authors of this paper predicting the resin properties (gelation point, crosslink density, volumetric expansion, glass transition temperature,…) based on the monomer chemical structure and properties as input and in good agreement with experimental data.^21^ Interestingly, these properties appeared to depend strongly on even slight modifications of the monomers. Compared to the previously used P-*p*PDA precursor for protection of anodized aluminum AA2024-T3 substrates, a benzoxazine based on 4-ethylphenol-*para*-phenylenediamine (4EP-*p*PDA) (Fig. 1) appeared to show different and promising properties compared to P-*p*PDA such as a substantial lower glass transition temperature (*T*_g_ = 220 °C and 150 °C for fully cured P-*p*PDA and 4EP-*p*PDA respectively) and this difference was explained by differences in density of H bonds between each of the fully cured benzoxazines.^21^

In this work, a laboratory synthesized benzoxazine precursor obtained from 4-ethylphenol-*para*-phenylenediamine (4EP-*p*PDA) has been applied on sulfo-tartaric anodized AA2024-T3 substrates. Compared to the previously studied P-*p*PDA resin,^20^ the new molecular structure of the 4EP-*p*PDA resin leads to a quite different behavior and more specifically at high temperatures. Indeed, this new precursor presents a much lower viscosity when heated above its melting point, promoting the mobility of benzoxazine molecules and, therefore, favoring the impregnation of the anodic layer porous structure by the resin. In order to evaluate the molecular mobility and impregnation of the resin during the curing step, the surface texture of the studied systems has been compared prior and after the curing process by Scanning Electron Microscopy-Field Emission Gun (SEM-FEG) observations completed with Glow-Discharge Optical Emission Spectroscopy (GDOES) depth profiling.

Electrochemical properties of impregnated samples have also been investigated by EIS. Thanks to these characterizations, the importance of the curing step in the achievement of high and durable barrier properties has been assessed.

Finally, the impact of the curing process duration and temperature on the achieved barrier properties and their durability has been investigated in order to obtain a thermal curing process compatible with the dependence of the mechanical properties of aeronautical aluminum substrates on thermal treatment at high temperatures.^22^

## 2 Experimental

### 2.1 Preparation of 4-ethyl-3,4-dihydro-2*H*-1,3-benzoxazine (4EP-*p*PDA)

**Fig. 2:**
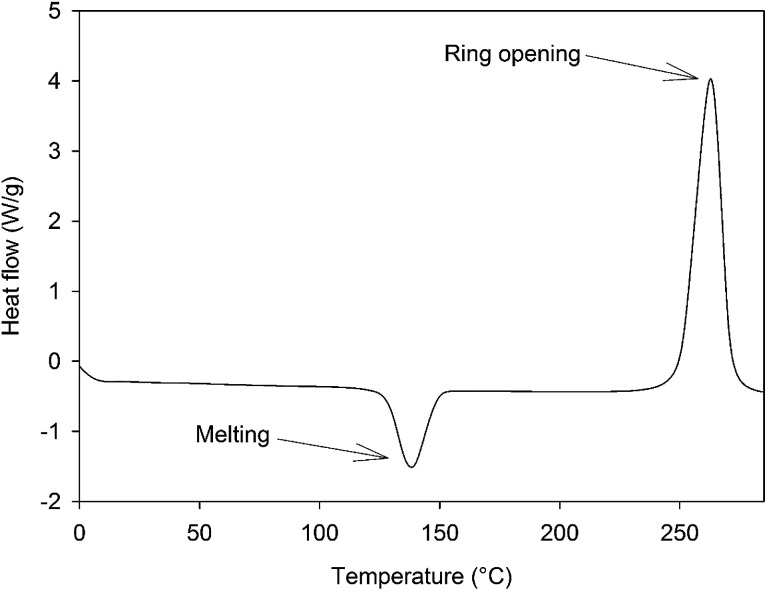
DSC curve of the 4EP-*p*PDA monomer from room temperature up to 280 °C at 10 °C min^−1^.

The synthesis of 4EP-*p*PDA has been adapted from a procedure in bulk reported elsewhere by Ishida *et al.*^23^ 4-Ethylphenol 22.82 g (1.9 × 10^−1^ mol) and *p*-phenylenediamine 10 g (9.25 × 10^−1^ mol) were mixed in a beaker with mechanical stirrer at 120 °C until a homogeneous liquid is obtained. Then, paraformaldehyde in excess 12.73 g (4.07 10^−1^ mol) was rapidly added under vigorous stirring to prevent bubbling from the rapid decomposition of paraformaldehyde into formaldehyde. The resulting mixture was reacted for 7 additional minutes under continuous stirring. The crude reaction product was dissolved in refluxing ethanol (about 500 mL) and the precursors of the resin were precipitated upon cooling. The resulting precipitate was collected, filtered and abundantly washed with cold ethanol. Then it was dried under vacuum at 140 °C for 15 minutes. A vitrified yellow resin was obtained (weight yield about 62%). The 4EP-*p*PDA precursors were characterized by a *T*_m_ of about 135 °C and a polymerization enthalpy of 74 kJ mol^−1^ occurring at a *T*_peak_ of 260 °C as displayed on Fig. 2.

### 2.2 Preparation process of the samples

The selected aluminum substrate was AA2024-T3. Details of its chemical composition are shown in Table 1.

**Table 1:** Elementary chemical composition in weight percent of used aluminum alloys

Alloy	Cu	Fe	Si	Mn	Mg	Cr	Zn	Ti	Others
2024-T3	3.8–4.9	0.5	0.5	0.3–0.9	1.2–1.8	0.1	0.25	0.15	0.15

Aluminum samples (60 mm × 45 mm × 1 mm) were treated prior to anodizing in order to enable the formation of a homogeneous oxide layer. The different steps are: degreasing in acetone, etching in 1 M NaOH at 40 °C for 1 min, desmutting in Turco® Liquid Smut-Go NC Deoxidizer at room temperature for 15 s with rinsing in deionized water between each step.

Anodizing was carried out in a sulfo-tartaric bath, using the following concentrations: 40 g L^−1^ H_2_SO_4_ + 80 g L^−1^ C_4_H_6_O_6_. The bath temperature was controlled using an outer water flow with set temperature. A fixed anodizing potential difference was applied using a Laboratory Power Supply EA Elektro-Automatik PS-2016-100. Based on previous research,^20^ anodizing parameters have been fixed as follows: bath temperature at 40 °C and applied potential difference at 10 V for 25 minutes. These settings are close to those commonly used in the industry.^25^ The resulting oxide layers have a total thickness of about 3–4 μm.

After pretreatment and subsequent anodizing, samples were rinsed again in deionized water, dried with pulsed air and directly coated with an organic solution of benzoxazine precursor in chloroform. This solution was obtained by dissolving the laboratory-synthesized 4EP-*p*PDA benzoxazine precursor (Fig. 1) in ≥99% stabilized GPR RECTAPUR® chloroform at room temperature, using manual stirring, at a concentration of 10 wt%. This solution was then applied on substrates by depositing 1 mL on the samples and then spin coated at 1000 rpm for 20 s.

After deposition and drying of the precursor coating at room temperature, thermal curing was carried out stepwise to obtain a cross-linked polymer by several thermal curing steps: first, successively at 170 °C for 60 min, at 190 °C for 120 min and, finally, at 210 °C for 60 min. The samples were thermally treated in a Heraeus Instruments LUT 6050 oven in horizontal position to avoid outflow and loss of benzoxazine during this treatment. Performing a progressive temperature increase with successive steps after melting is necessary in order to avoid premature gelation of the resin, limiting the mobility of the monomers and so the formation of a consistent 3D network. As curing goes on, the molecular mobility is progressively reducing and, therefore, the highest curing temperatures (190 °C and 210 °C) are required for providing the necessary mobility to the molecules for achieving a consistent 3D network. At the end of the heating cycle, samples were let to slowly cool down to room temperature. This curing cycle leads to a maximum crosslinking degree of the resin as shown on DSC curves (no residual enthalpy) and will be considered as the “full curing” process in this paper. Other thermal treatments, at lower curing temperatures, were also performed and will be detailed later.

### 2.3 Characterization

#### 2.3.1 Rheological analysis at high temperature

The rheological properties of the 4EP-*p*PDA monomer at high temperature have been characterized using an ARES-LS2 equipment from TA instruments. Measurements were performed in dynamic mode with a parallel plate geometry (diameter of the plates = 40 mm, spacing of 0.5 mm between the plates). The applied deformation was 10% at a frequency of 1 Hz.

#### 2.3.2 Crosslinking degree estimation after curing

DSC measurements (Q200 from TA Instruments) under N_2_ (50 mL min^−1^) with a temperature ramp of 10 °C min^−1^ were performed on benzoxazine samples in order to observe the evolution of the heat flow of the material during the temperature ramp. The polymerization peak was integrated in order to estimate the enthalpy associated with the crosslinking. An indium standard was used for calibration.

The crosslinking degree *χ* was evaluated according to the eqn (1):1
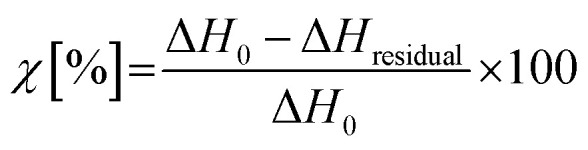
where Δ*H*_0_ corresponds to the total reaction enthalpy measured on non-cured samples and Δ*H*_residual_ corresponds to the enthalpy measured after a given curing cycle.

#### 2.3.3 Textural analysis of anodized and coated samples

Anodized and coated samples were observed using SEM-FEG (Hitachi SU8020 with cold cathode). In order to avoid textural changes, no metal layer was coated on the samples prior to observation. Cross section observations were performed by cutting samples using cryogenic breaking: after one-minute immersion in liquid nitrogen, samples were clamped and bent to rupture. Using Image J 1.48v software, the thickness of the polymer layers has been measured on SEM-FEG micrographs.

#### 2.3.4 Glow discharge optical emission spectrometry

Composition depth profiles of the samples were determined by means of Glow Discharge Optical Emission Spectroscopy (GDOES) using a Horiba Jobin Yvon HR-profiler instrument at an Ar pressure of 650 Pa and an applied power of 40 W, obtained through a 14.56 MHz Rf-Generator. The GDOES instrument was equipped with a standard 4 mm diameter anode and a 0.5 m Runge–Paschen polychromator with 28 acquiring channels. The Polychromator is purged in Nitrogen at least 24 h before the measurements. For this work, only Al, O, N, C and S signals have been acquired. The measurement method has been previously calibrated, in order to obtain quantitative profiles, using a sputtering rate method and thus constructing calibration curves by means of 21 SUS (Setting Up Samples) and CRM (Certified Reference Materials). The instrument and results have been controlled and elaborated by means of the quantum XP software.

#### 2.3.5 Electrochemical properties and modeling

A conventional three-electrode cell was used for the electrochemical tests. The working electrode was the investigated sample (exposed area of 7.07 cm^2^), the counter electrode was a platinum plate and all potentials were measured with respect to an Ag/AgCl/Satured KCl (+0.197 V *vs.* SHE) reference electrode. The cell was placed in a Faraday cage in order to minimize external electromagnetic interference on the system. The impedance measurements were carried out over frequencies ranging from 100 kHz to 10 mHz, at ambient temperature. The impedance spectra were acquired by using a potentiostat coupled with a frequency response analyser (Parstat 2273 from Ametek), computer-controlled with Powersuite® software. Electrochemical impedance measurements were performed after different immersion times in 0.1 M NaCl solution on the different systems. The signal amplitude was 30 mV rms. Three samples of each type have been characterized in order to check the reproducibility of the EIS results. The presented EIS spectra correspond to the representative behavior of each type. Impedance measured data have been fitted using equivalent electrical circuits. The impedance values of electrical components have been iterated to fit experimental impedance data using a fitting software: ZSimpWin 3.50.

## 3 Results and discussion

### 3.1 Rheological properties of the 4EP-*p*PDA resin at high temperature

**Fig. 3:**
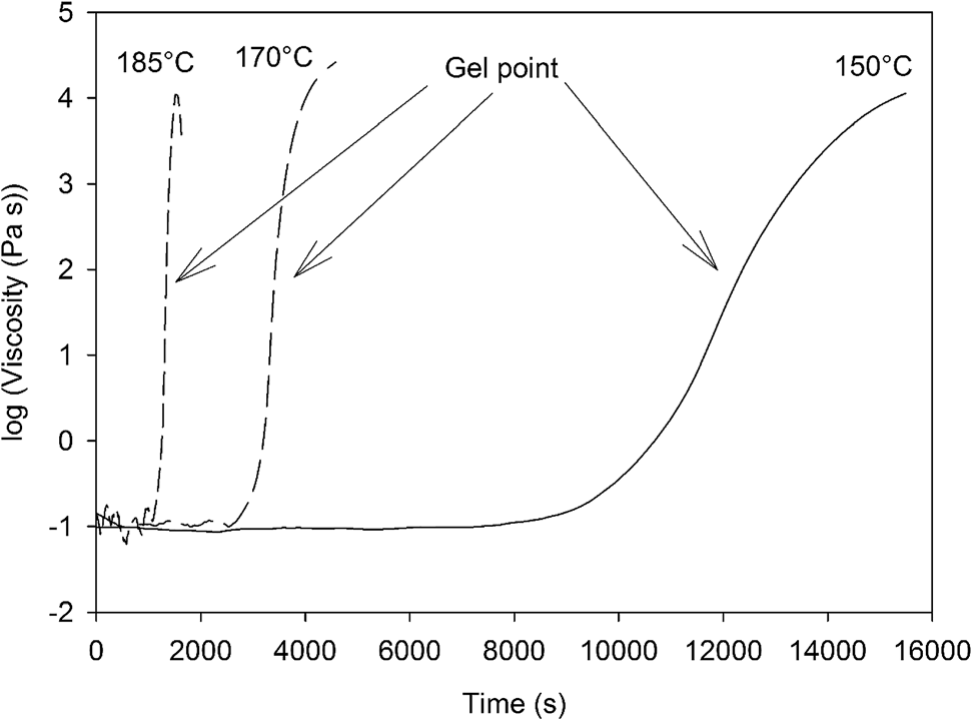
Evolution of the viscosity of the 4EP-*p*PDA over time with different applied isotherms.

In order to assess the viscosity of the synthesized benzoxazine at high temperature as well as the relation between the gelation time and the curing temperature, the 4EP-*p*PDA resin has been characterized by rheological measurements at 1 Hz at different temperature isotherms above the melting point of the molecule (∼135 °C). The evolution of the viscosity with time is displayed on Fig. 3.

From the evolution of the viscosity, it can clearly be observed that the resin has a very low viscosity when heated above its melting temperature. The noisy behavior at the beginning of the measurements is related to the very low viscosity of the resin, close to the limits of the instrumentation and of the geometry (parallel plates) of the rheological measurement. Indeed, the viscosity values at the beginning of the measurement are of the order of 0.1 Pa s for all isotherm temperatures.

However, after a given time and depending on the temperature, a very rapid increase of the viscosity occurs, associated with the gel point of the material. The delay to reach this gel point is directly related to the isotherm temperature as summarized in Table 2.

**Table 2:** Measured gel point times obtained from viscosity measurements at different isotherm temperatures

Isotherm temperature (°C)	Gel point time (min)
150	240
170	70
185	25

Higher isothermal temperatures lead to shorter gelation times, which can be attributed to the fact that at higher temperatures the ring opening reaction is favored and therefore the formation of a cohesive 3D network accelerated.

The curing cycle selected for the standard curing of the 4EP-*p*PDA resin is presented in Table 3. The progressive increase of the temperature from 170 °C up to 210 °C allows to provide mobility to the reactive monomers, carefully avoiding the risk of premature gelation of the material at the lower temperatures. A maximal cross-linking density can then be achieved, attributed to a maximum crosslinking degree close to 100%.

**Table 3:** Successive dwells of the full curing cycle of 4EP-*p*PDA

Temperature of the dwell (°C)	Duration of the dwell (min)
170	60
190	120
210	60

### 3.2 Impregnation of the oxide layer by the resin during the curing process

**Fig. 4:**
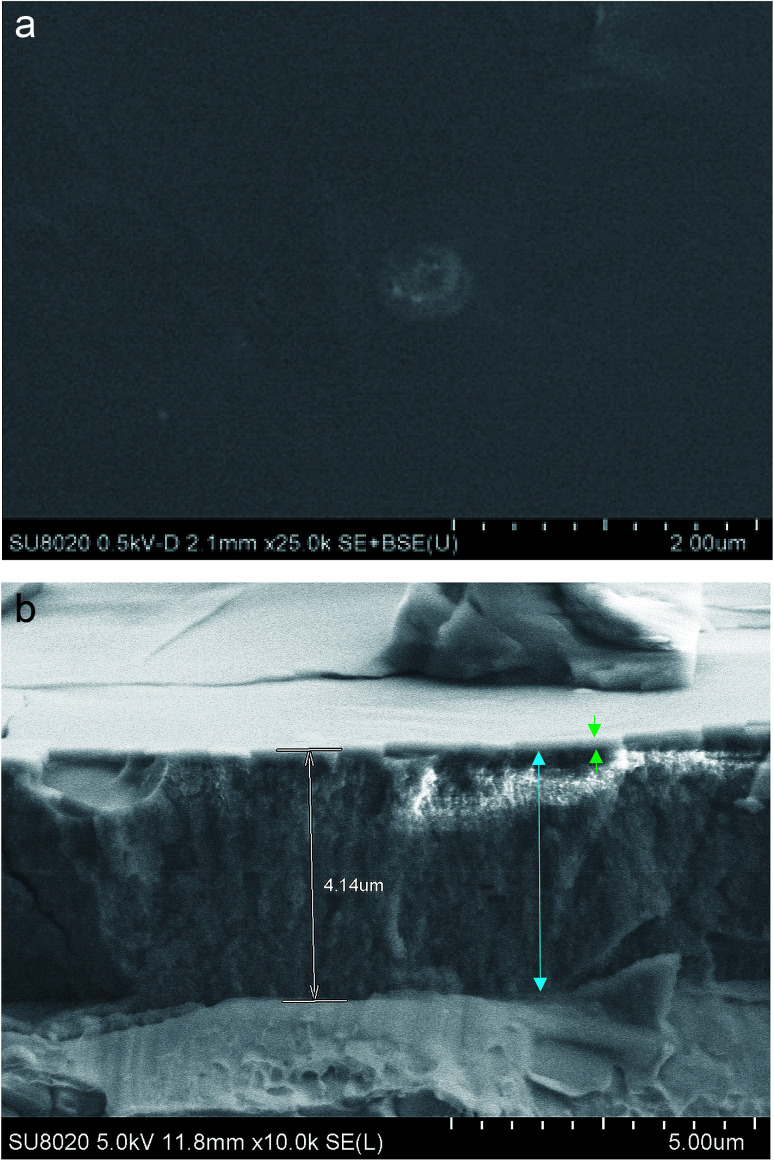
SEM-FEG micrographs of the (a) surface and the (b) cross-section of the oxide layer (blue arrow) on AA2024-T3 obtained from anodizing (10 V, 25 min, 40 °C) and spin coated with a non-cured 4EP-*p*PDA precursor (green arrow).

Fig. 4a and b show the surface and the cross-section respectively of an anodic film obtained on AA2024-T3 and spin coated with the 4EP-*p*PDA benzoxazine precursor, prior to curing. In Fig. 4a, it can be observed on the surface that the resin deposit on top of the oxide layer is forming a smooth and uniform coating. From the cross-section observation (Fig. 4b), the thickness of this organic layer can be estimated to be approximately 0.1 μm, while the thickness of the anodic layer is about 4.0 μm, the latter being comparable to thicknesses usually reported in the literature^24^ and homogeneously following the roughness of the substrate. The morphology of the oxide layer has a rather sponge-like porous structure, quite different from the theoretically predicted structure of anodized aluminum consisting of columnar pores perpendicular to the metal surface organized following an hexagonal pattern.^25^ The latter “ideal” structure is obtained by TSA of pure and polished aluminum.^26^ However, in this paper, the substrate of interest is an industrial class of AA2024-T3, an alloy with a composition different from the ideal case of pure aluminum. The particular sponge-like morphology has already been observed in the literature and was attributed to the generation of oxygen gas at copper-rich sites during anodizing^20,27^ generating these particular pores.

**Fig. 5:**
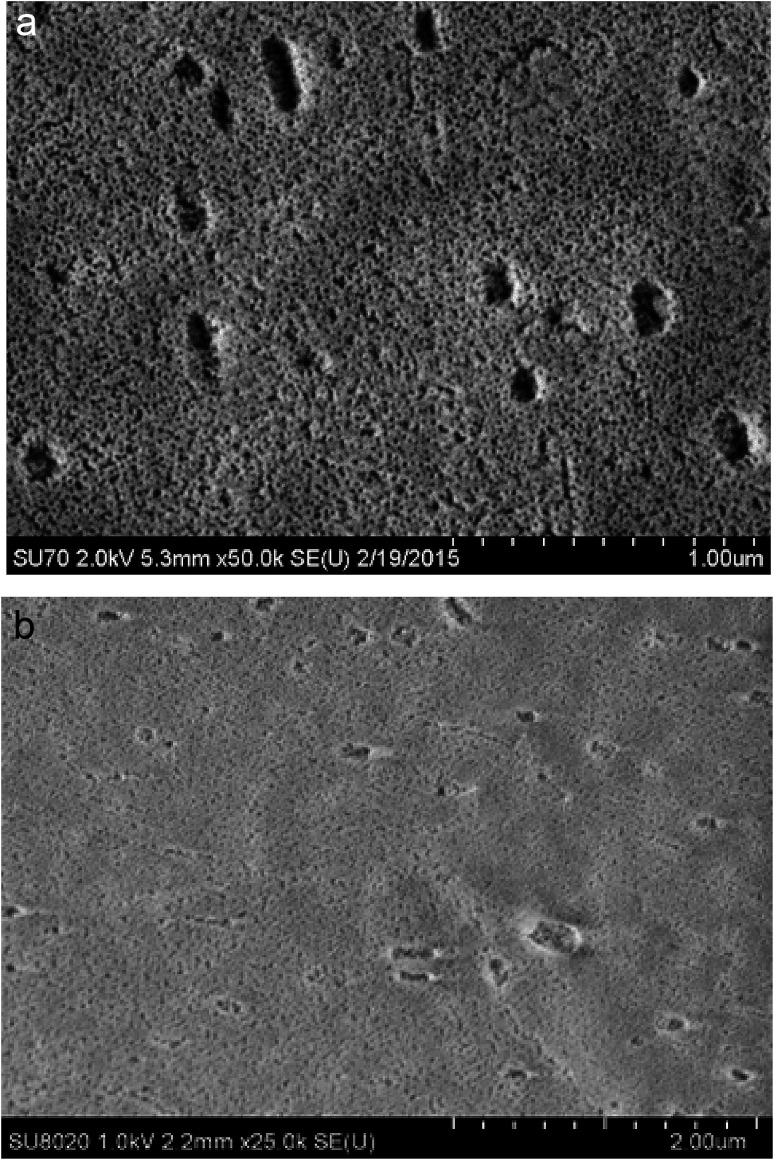
SEM-FEG micrographs of the surface of the oxide layer on AA2024-T3 obtained from anodizing (10 V, 25 min, 40 °C) (a) after anodizing and (b) after anodizing and spin coated with 4EP-*p*PDA resin fully cured.

Fig. 5 shows the surface of an anodic film obtained on AA2024-T3 and spin coated with the 4EP-*p*PDA benzoxazine precursor, after standard curing from 170 °C up to 210 °C for 4 hours. This SEM-FEG micrograph shows that, after curing, the surface porosity of the porous oxide layer appears again, as before application of the coating.

A plausible interpretation of this observation is that impregnation of the porous oxide layer by the benzoxazine resin is taking place, mainly during the curing process. Indeed, the low viscosity of the 4EP-*p*PDA molecule starting from the fusion temperature might offer the opportunity to the resin precursor to freely flow through the oxide layer, impregnating its porous structure during this part of the curing cycle. Consequently, the top layer would be soaked within the pores of the anodic layer, leading to a reappearance of the pores at the surface.

**Fig. 6:**
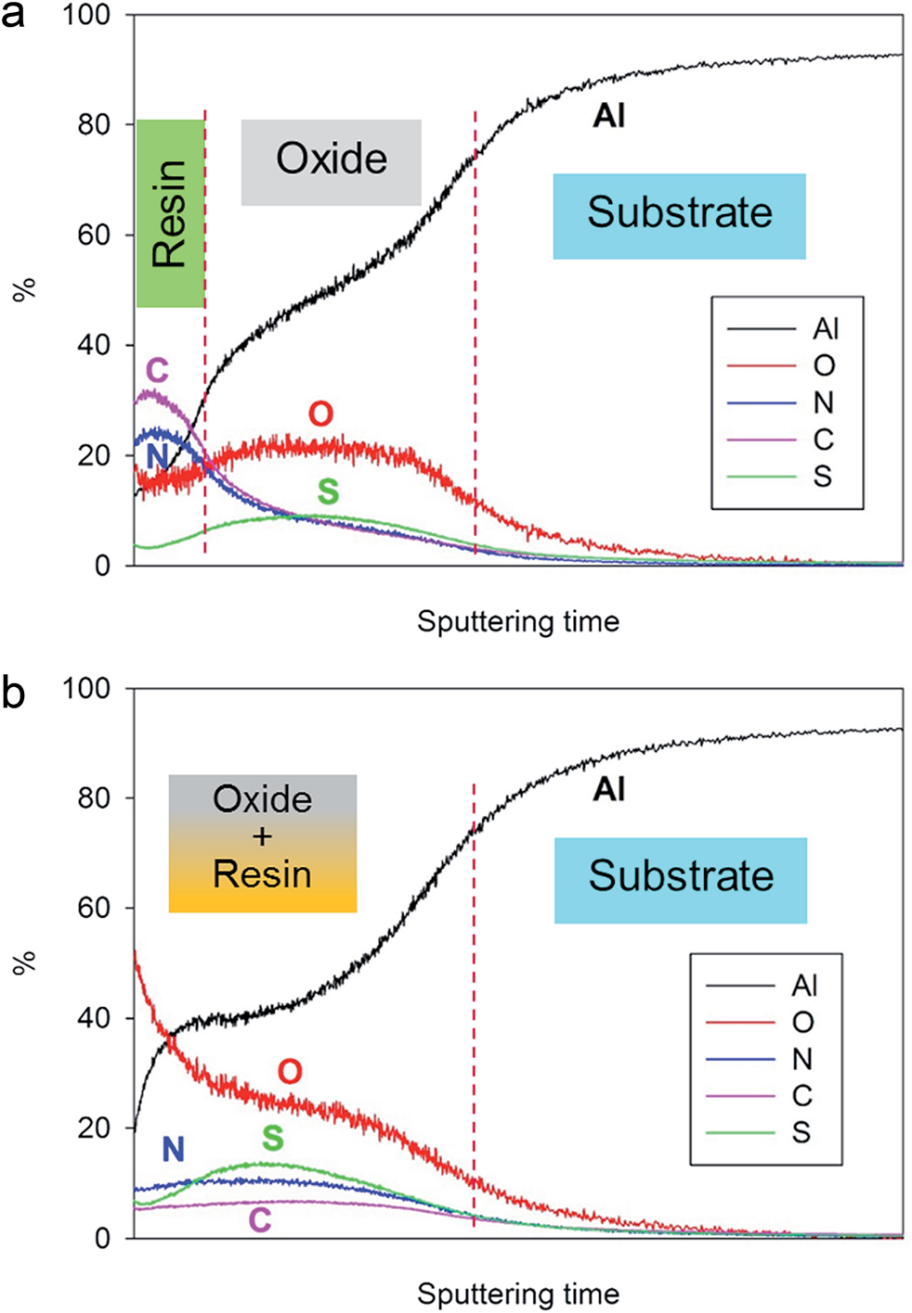
GDOES depth profiles of (a) non-cured and (b) fully cured 4EP-*p*PDA resin applied on anodized AA2024-T3 (10 V, 25 min, 40 °C).

In order to confirm this hypothesis, GDOES profiling has been performed on both non-cured and fully cured 4EP-*p*PDA resin deposited on anodized AA2024-T3 substrates. The chemical composition profiles of the analyzed samples are displayed in Fig. 6a and b. In these graphs, the evolution of the composition over the sputtering time – which is directly related to the sputtered depth – is followed to track the different parts (organic and inorganic) of the system. As the sputtering rate can be significantly influenced by the composition of the layers or by the cross-linking density of the organic compounds, the sputtering time has not been converted in thickness of the analyzed layers. The carbon and the nitrogen signals can be attributed to the resin, while the oxygen and the sulfur (coming from the anions of the anodizing bath) are mainly associated with the anodic layer. Aluminum is present in both the oxide layer and the metallic substrate. Considering the significant roughness of the samples, the GDOES profiles have been interpreted qualitatively.

By comparing the two depth profiles in Fig. 6a and b, a significant difference between the non-cured sample and the cured one can be noticed. Indeed, in the case of the non-cured sample, high carbon and the nitrogen signals appear at the very beginning of the sputtering, in accordance with the resin top layer observed by SEM-FEG (Fig. 4a) and their ratios with respect to the oxygen signal are significantly higher than 1 at the surface and decreasing from the surface towards the aluminum substrate. The presence of carbon and nitrogen in the anodized part of the graph might be attributed to a possible partial penetration of the organic coating solution in the anodic layer during spin coating and subsequent drying.

Concerning the fully cured system, the high C and N signal on top of the surface is no longer observed and carbon and nitrogen atoms are more homogeneously distributed throughout the anodic layer, confirming the oxide impregnation and the coexistence of both organic and inorganic parts at the same sputtering depth. Both C and N signals are lower in intensity than observed before curing and this can be attributed to the network formation during curing decreasing the sputtering efficiency, as has also been observed during ToF-SIMS analysis of P-*p*PDA benzoxazine coatings^19,28^ The higher intensity of the oxygen signal at the top surface after curing can probably be explained as a result of oxidation reactions occurring during the thermal curing process in air.

These observations suggest that the organic coating on top of the surface before curing, has penetrated within the porous structure during curing and, consequently, leaving the layer close to the surface uncoated and porous as was observed in Fig. 5. These results are in line with GDOES results reported in the literature on sol–gel coatings applied on similar TSA aluminum substrates and where similar profiles were obtained depending on the penetration of the sol–gel within the porous structure.^29^ The 4EP-*p*PDA can therefore be considered as a sealant rather than a coating, the sealing occurring during the curing process, inducing the melting and infiltration of the resin within the porous anodized layer. It's interesting to notice that this surface porosity and high area of interaction could offer a great mechanical anchoring for organic coatings, paints or varnishes to be applied on top of it depending on the kind of emphasized application. This kind of coating fully differs from a previously reported P-*p*PDA benzoxazine layer coated and cured on TSA anodized AA2024-T3 according to the conditions described by the authors in a previous paper^20^ and where, due to the much higher viscosity during curing, no infiltration and sealing of the anodized layer occurred but, instead, the coating was anchored on top of the anodized layer.

### 3.3 Electrochemical properties of 4EP-*p*PDA applied on anodized aluminum

#### 3.3.1 EIS performed on non-cured and fully cured systems

**Fig. 7:**
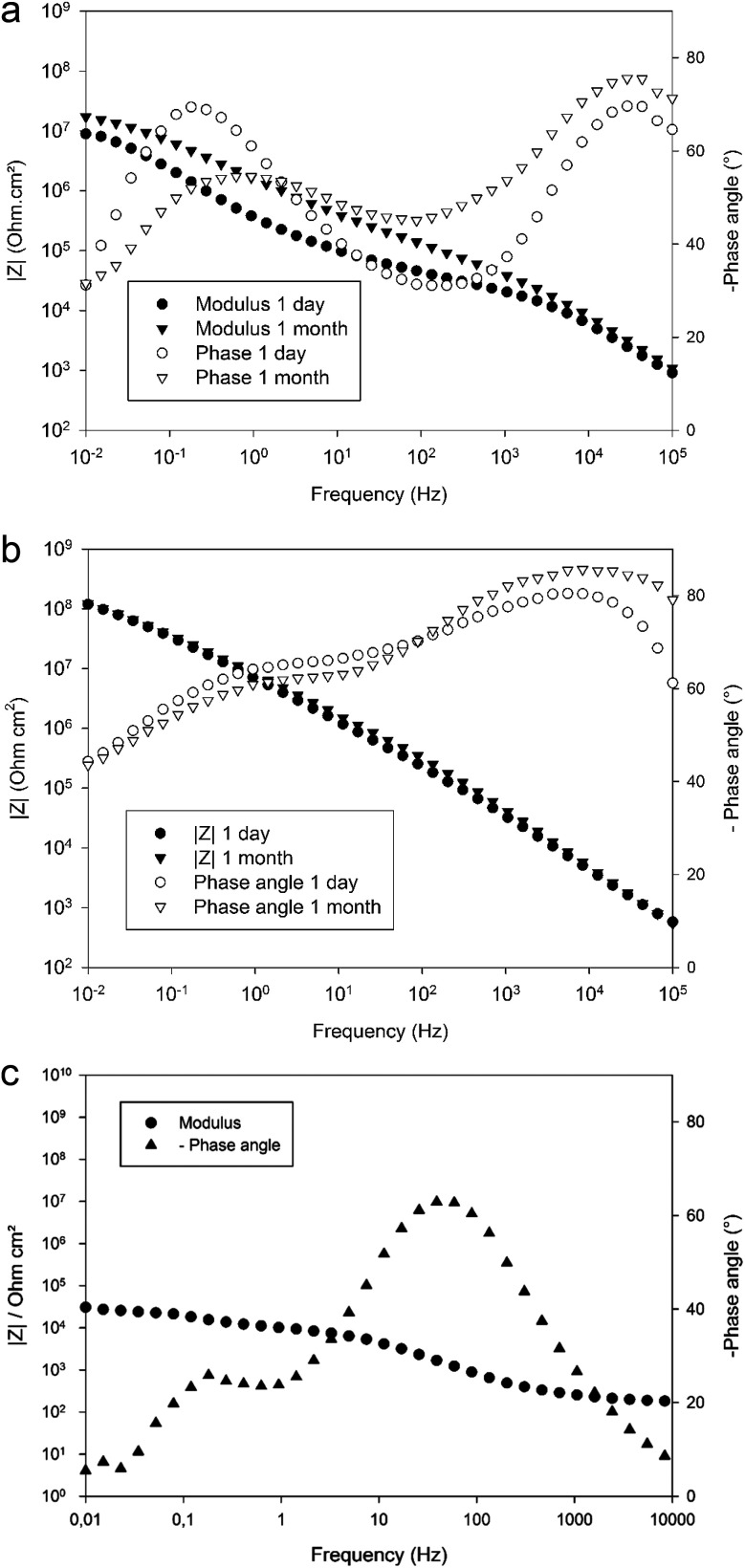
Evolution of the Bode impedance modulus and phase angle obtained for (a) non-cured and (b) fully cured 4EP-*p*PDA applied on anodized AA2024-T3 over one month of immersion in 0.1 M NaCl electrolyte – (c) Bode impedance modulus and phase angle after 24 h of immersion obtained for anodized AA2024-T3 with no 4EP-*p*PDA for comparison purpose.

Fig. 7a and b compare the evolution of the impedance spectrum over immersion time in 0.1 M NaCl electrolyte obtained from EIS on non-cured (Fig. 7a) and fully cured (Fig. 7b) 4EP-*p*PDA spin coated samples on anodized AA2024-T3. A significant difference can be observed already at the beginning of the immersion period. Non-cured systems show two fully separated time constants in the phase angle plot – one in the high frequency range (≥1 kHz) and one in the low frequency range (≤0.1–1 kHz). The absolute value of the maximum phase angle values for both time constants are each lower than 70°. In terms of impedance modulus, its value reaches a maximum value of 10^7^ ohm cm^2^ in the lowest frequency decade. Moreover, comparing Fig. 7a and b, it can be observed that the impedance spectrum of the non-cured system is significantly evolving over one month of immersion, whereas it remains almost unchanged over the same period after curing. Indeed, Fig. 7a shows how the phase angle spectrum of the non-cured system is changing with time, and how the impedance modulus is increasing at all frequencies. This increase of impedance modulus can be attributed to the partial hydration of the porous part of the oxide layer, progressively influencing the electrochemical properties as has already been reported in the literature on anodic layers.^30^ Indeed, though the substrate appears to be fully covered by the organic coating as observed on Fig. 3b, the latter might be insufficiently consolidated to impede electrolyte infiltration through this non-cured coating subsequently reaching the oxide layer. As a consequence, the oxide layer is left directly exposed to the electrolyte, explaining the resistive behavior at the beginning of the immersion period. Hydration of the exposed oxide could then occur with time, explaining the observed increase of impedance. Such an evolution shows the unstable behavior of the electrochemical properties of the coated substrate before curing. However, one should notice, this system still offers higher barrier properties compared to anodized AA2024-T3 which displays a modulus at low frequency between 10^4^ and 10^5^ ohm cm^2^, at least two decades lower than the coated samples, after 24 h of immersion in the saline electrolyte, as shown in Fig. 7c.

After thermal curing, the electrochemical properties of the fully cured system appear to be much more stable with time, as shown in Fig. 7b. In the phase angle diagram, two time constants can again be distinguished. However, since resin and oxide materials are now coexisting as a hybrid organic/inorganic layer, the two time constants cannot be simply attributed to either one of them. Moreover, the two time constants are much wider and more overlapping than in the case of the non-cured system. In addition to this greater stability, both modulus and phase angle exhibit higher values: the phase angle absolute value is always higher than 40° in the whole frequency range, and the impedance modulus reaches 10^8^ ohm cm^2^ in the lowest frequency decade. Comparing the evolution of the EIS spectra of non-cured and fully cured systems over immersion time in 0.1 M NaCl, it can be concluded that the curing process is the key step for obtaining high and durable barrier properties. As was previously proposed in this paper based on GDOES experiments, during the curing step, the benzoxazine resin impregnates the porous oxide layer forming a hybrid organic–inorganic layer exhibiting highly capacitive electrochemical properties. The obtained barrier properties are comparable or better than those observed for anodic layers of comparable nature and thicknesses sealed in boiling water^30,31^ or impregnated with sol–gel materials.^29,30^ Moreover, considering the high surface porosity of the fully cured system (Fig. 5), this could offer good anchoring conditions for top layers applied on top of it. Therefore, the cured system is a promising candidate for reaching high barrier properties in order to protect painted or varnished parts from corrosion. Finally, as will be shown in part 3.3.2, partial curing of the 4EP-*p*PDA coating leads to even better barrier properties which could also improve the interface between the benzoxazine primer and applied epoxy-based paint through chemical reaction.^32^

#### 3.3.2 Influence of the curing conditions on the electrochemical properties of a 4EP-*p*PDA coating

The study of the influence of the decrease of the temperature and duration of the curing cycle has been motivated by two reasons.

First, in the framework of aeronautical applications, it is necessary to keep in mind the thermal sensitivity of the substrate of interest. Indeed, AA2024-T3 aluminum alloys cannot withstand temperatures higher than 170 °C for a long time without losing their high mechanical properties. This mechanical failure is attributed to the modification of hardening precipitates from *θ*′ phase, having a semi-coherent interface with the aluminum matrix, to *θ* phase having an incoherent interface with the matrix.^22^ The 4 hours long and high temperature (up to 210 °C) full curing cycle of the 4EP-*p*PDA is not compatible with the thermal sensitivity of the AA2024-T3 substrate. Secondly, the authors have shown in a previous paper^20^ that the complete curing of a P-*p*PDA benzoxazine could lead to a premature loss of barrier properties through the creation of resistive pathways within the cured resin.

**Fig. 8:**
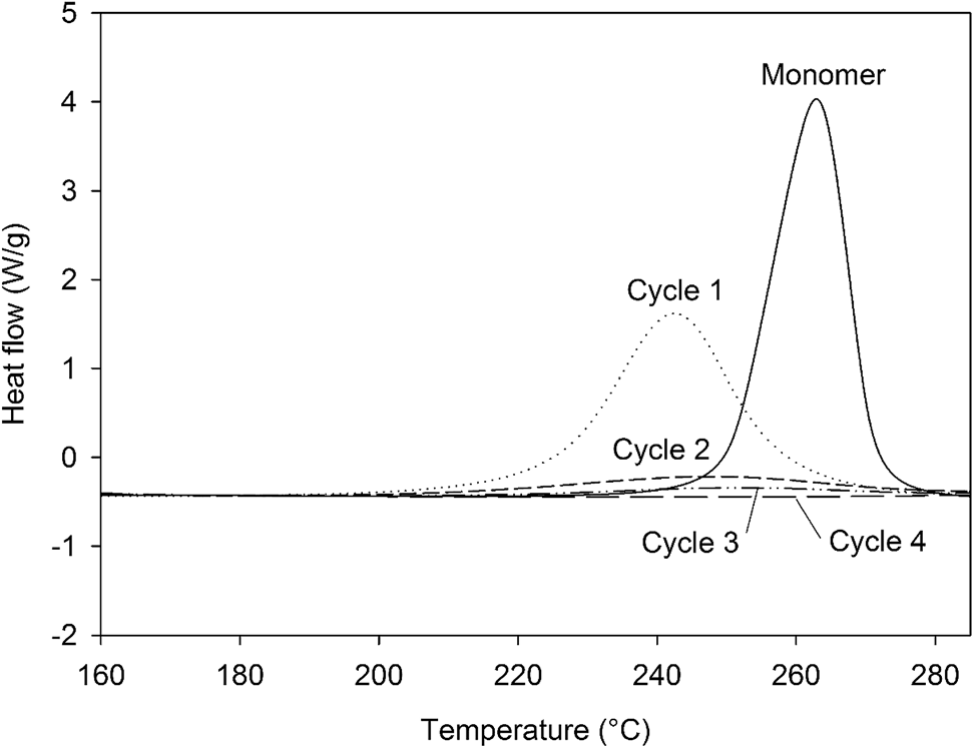
DSC curves (160 °C to 285 °C, 10 °C min^−1^) of the residual enthalpy peaks of the 4EP-*p*PDA cured according to different curing cycles.

In order to assess the impact of the different steps of the curing process, a formulation of 4EP-*p*PDA in chloroform (10 wt%) has been applied on anodized AA2024-T3 substrates and cured according to the 4 different cycles presented in Table 4, showing also the corresponding crosslinking degrees, which have been estimated from the residual enthalpy values measured by DSC (Fig. 8). The complete crosslinking provided by the full curing cycle, here named Cycle4, is here evidenced. It is worth to notice that the peak maxima of partially cured samples are shifted to lower temperatures compared to the pristine monomer. This phenomenon illustrates the autocatalytic nature of the polymerization reaction, as free phenol groups are formed which can accelerate the ring-opening process.^33,34^ By removing the step at 210 °C (Cycle3), the impact of this final step at the highest temperature (leading to the 100% crosslinking) can be isolated. The impact of the crosslinking advancement at the same curing temperature can also be studied from the comparison between Cycle2 and Cycle3, using different durations for the step at 190 °C. Finally, Cycle1 allows to evaluate the properties of the system where the resin is poorly cross-linked and did not reach the gelation point.

**Table 4:** Curing cycles used to cure 4EP-*p*PDA applied on anodized AA2024-T3 substrates with corresponding crosslinking degrees estimated from DSC measurements

Cycle designation	170 °C	190 °C	210 °C	Crosslinking degree (%)
Cycle4 (=full curing)	60 min	120 min	60 min	100
Cycle3	60 min	120 min	—	96
Cycle2	60 min	60 min	—	87
Cycle1	60 min	—	—	16

**Fig. 9:**
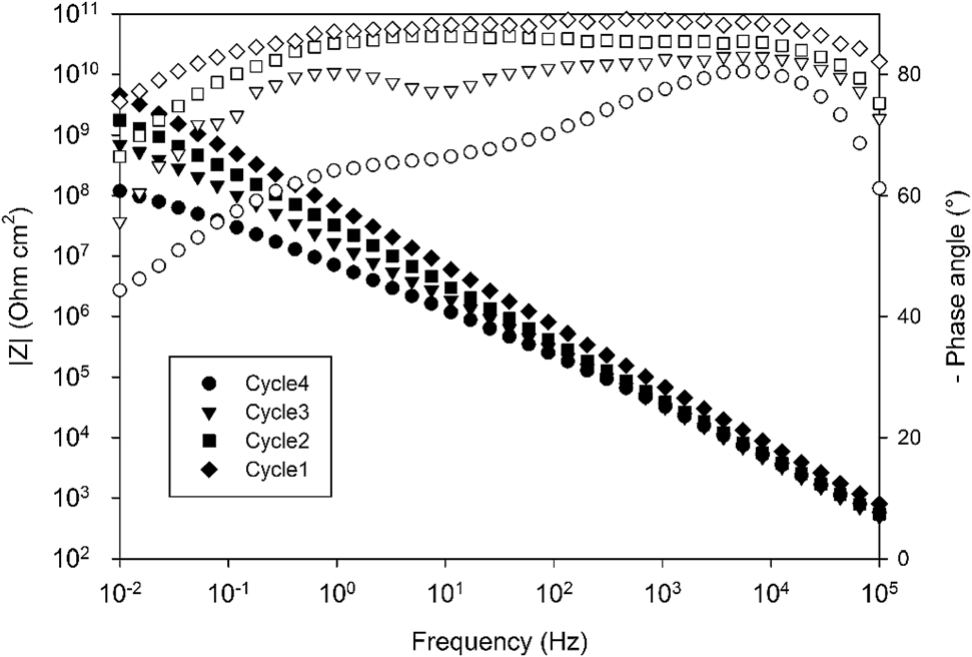
Comparison of the Bode impedance modulus and phase angle diagrams obtained for a 10 wt% solution of 4EP-*p*PDA applied on anodized AA2024-T3 and cured according to different curing cycles after 24 h of immersion in 0.1 M NaCl.

The electrochemical properties of the obtained systems have been characterized by EIS over one month of immersion in 0.1 M NaCl electrolyte. Fig. 9 compares the EIS spectra obtained for a 10 wt% 4EP-*p*PDA solution applied on anodized AA2024-T3 and cured according to the four different cycles, after 24 h of immersion in the saline electrolyte. Surprisingly, from this comparison it is clear that the shorter the cycle and the lower the temperature, the higher the barrier properties. Indeed, Cycle1 samples, corresponding to only one hour curing at 170 °C, exhibit the highest impedance modulus at low frequency (around 10^10^ ohm cm^2^) and the most capacitive behavior among all samples. On the other hand, samples cured according to the full standard cycle “Cycle4” present the least capacitive behavior and the lowest impedance modulus (around 10^8^ ohm cm^2^).

**Fig. 10:**
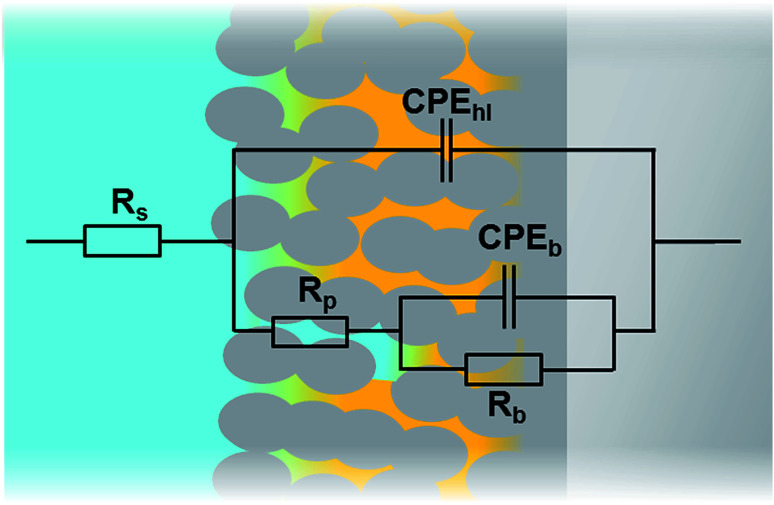
Equivalent electrical circuit used for fitting the impedance data of the different systems.

**Fig. 11:**
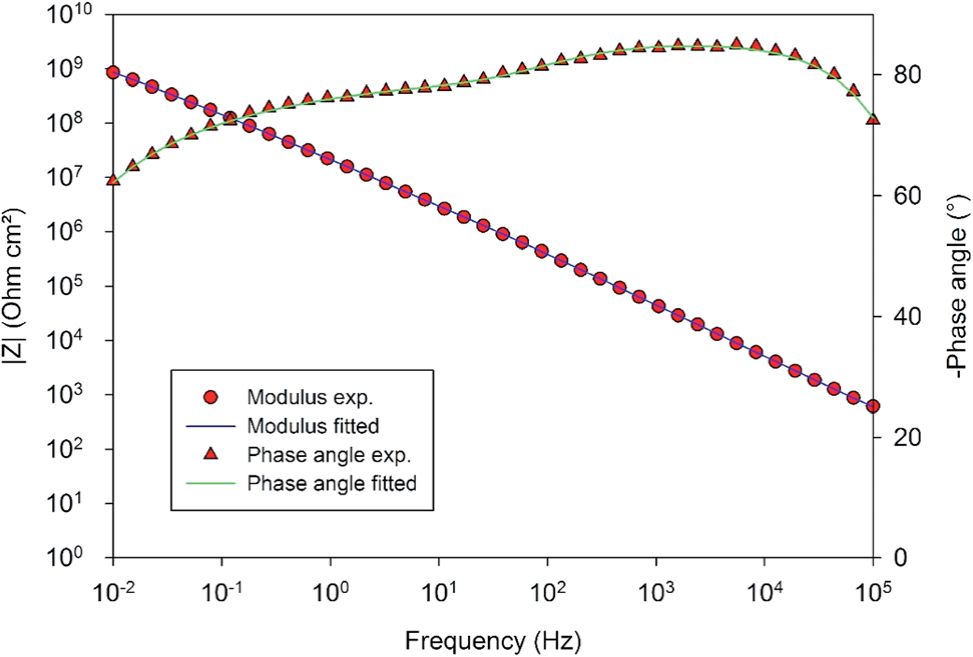
Comparison of the EIS experimental and fitted data in the example of the 10 wt% solution of 4EP-*p*PDA applied on anodized AA2024-T3 after one month of immersion in 0.1 M NaCl.

In order to compare the different systems more quantitatively and to follow their evolution over immersion time in the 0.1 M NaCl electrolyte, the impedance data have been fitted using the Equivalent Electrical Circuit (EEC) displayed in Fig. 10. Since all systems exhibit similar electrochemical behaviors with the presence of two time constants, this EEC has been applied to fit the impedance data of all systems. Constant Phase Elements have been used instead of pure capacitances in order to reflect the complexity and the non-ideal character of the system. In the literature, the impedance of a constant phase element is written as *Z*_Q_ = 1/(*Q*_0_(i*ω*)^*n*^) where *Q*_0_ is the CPE admittance and the CPE exponent *n* is the frequency dispersion factor varying from 0 to 1. If *n* = 0, the CPE behaves as a resistor. If *n* = 1, the CPE is a pure capacitance.^35^*R*_s_ is the resistance of the electrolyte, CPE_hl_ refers to the non-ideal capacitive behavior of the overall hybrid layer, *R*_p_ is the resistance of the resistive pathways going through the hybrid layer, and CPE_b_ and *R*_b_ are the non-ideal capacitive behavior and the resistance of the bottom of the hybrid layer, which is supposed to be a combination of the oxide barrier layer resulting from the anodizing process and of some organic material which has reached the bottom of the porous layer. The exact EEC corresponding to this system is probably more complex. However, because of the strong overlapping of the time constants and the limited frequency range of the EIS, this simplified model has been used. The fitting quality is very good, as shown in the example of the Fig. 11.

**Fig. 12:**
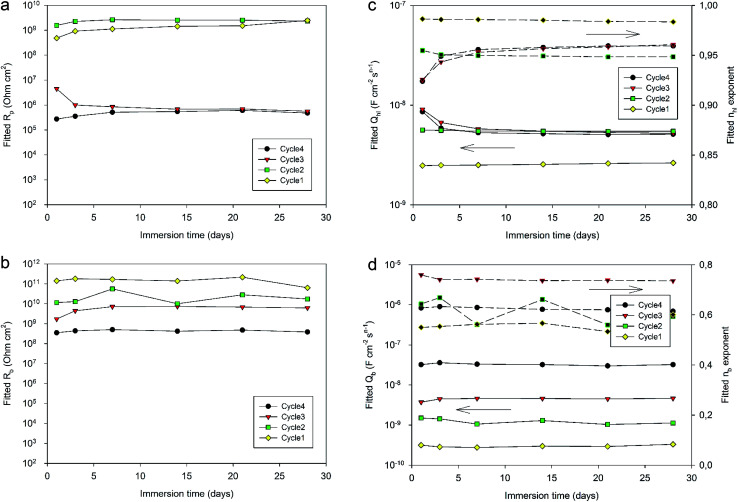
Evolution of the fitted parameters (a) *R*_p_, (b) *R*_b_, (c) *Q*_hl_ and (d) *Q*_b_ (with corresponding *n* values in black) of a 10 wt% solution 4EP-*p*PDA applied on anodized AA2024-T3 and cured according to different curing cycles over one month of immersion in 0.1 M NaCl.

Fig. 12 displays the evolution of the different fitted parameters over one month of immersion for the four different curing cycles. All fitted parameters exhibit a very stable behavior. Durable barrier properties are then achieved for all curing cycles. However, significant differences can be observed depending on the applied curing cycle.

First, *R*_p_ values (Fig. 12a) obtained for Cycle4 and Cycle3 samples compared with values obtained for Cycle2 and Cycle1 samples are significantly different. Indeed, *R*_p_ values for shorter cycles appear to be almost 4 orders of magnitude higher than values obtained with Cycle3 and Cycle4 samples. These differences appear not to be simply correlated with the cross-linking degree measured by DSC characterization (Table 4). Otherwise, the *R*_p_ value of Cycle2 should be closer to Cycle3 and Cycle4 rather than Cycle1. The origin of the observed differences may be related to the molecular interactions and configurations generated during curing. Indeed, going stepwise from Cycle1 to Cycle4, as curing progresses, the samples are exposed to higher temperatures for progressively longer durations and, though the resin becomes more and more crosslinked, this does not appear to lead to a progressively better corrosion barrier protecting the substrate. This can be understood in terms of molecular interactions between monomers, oligomers or polymers, the proportion of each evolving as curing proceeds. Indeed, recently, MDS studies simulating and comparing the polymerization of P-*p*PDA and 4EP-*p*PDA have been published in the literature^21^ considering the condition of close contacts occurring between reactive atoms (4 to 8 Å) eventually leading to the formation of the Mannich bridge. As monomers are linked together in this way, chain branching occurs, progressively increasing in size as curing goes on and, finally, spanning the whole considered system. This percolating molecule is considered as the gel fraction, whereas all other chains constitute the sol fraction. The gel fraction contains structures such as dangling chains and loops, which do not participate to the elastic behavior of the resin. In the case of 4EP-*p*PDA, it has been shown that the resin starts to show an elastic behavior close to 40% of conversion, corresponding to the gel point. For lower conversion degrees, no percolating chains exist, and molecules are progressively ramifying as conversion progresses which is again expressed by a decrease of free molecules belonging to the sol phase and a progressive increase of dangling and loop chains. Above the gel point, the elastic contribution of the system increases as dangling and loop chains are progressively consumed, and free monomers completely disappear at 60% conversion. At 90% conversion the resin consists of fully crosslinked polymers and becomes completely elastic. The glass transition temperature (*T*_g_) of crosslinked polybenzoxazines is surprisingly high, ranging from 150 to 400 °C; this thermomechanical behavior has been related to extensive hydrogen bond linked networks, reducing chain mobility in the resins.^36^ In the case of polybenzoxazines, the elastic storage modulus reduction near *T*_g_ is much steeper due to a high degree of breaking of hydrogen bonds, compared to polymers whose thermomechanical stability do not depend on H-bonding.^37^ As a result, understanding the role of hydrogen bonds is a fundamental key towards comprehending the relationships between the structural topology and properties of benzoxazines, since they can be considered as additional weak crosslinking sites. As a consequence, it appears that the density calculated for 4EP-*p*PDA at room temperature for low conversion rates (20%) or highly crosslinked (80%) is comparable. In between, the density after curing, first, decreases and becomes lowest around the gel point and then increases again.

With respect to the systems studied in the framework of this paper, Cycle1 can be considered as leading to a poorly cross-linked (16%) but highly cohesive and dense cured resin mainly through the formation of H-bridges (OH–N and OH–O). Increasing the crosslinking, first decreases the density through a reduction of the number of H-bridges and, starting from the gel-point, again an increase of the density through cross-linking. For Cycle2, the cross-linking degree is 87%, of comparable density but with a higher elasticity and much less H-bridges. Above 90% of conversion, the system becomes completely elastic and H-bridges are no longer formed (Cycles 3 and 4). The electrochemical properties seem therefore to be closely related to the presence of H-bridges within the resin, leading to compact textures with good barrier properties. The complete rigidification of the gel leads to the lowest barrier properties appearing above 90% conversion (Cycles 3 and 4). A completely rigidified resin might introduce mechanical stresses within the system during the cooling step of curing, exacerbated at the resin alumina interface, which weakening is likely to explain the decrease of the *R*_p_ values. Moreover, at this point of research, we cannot exclude the occurrence of thermal degradation at the highest curing temperatures^38^ (Cycles 3 and 4) which are not taken into account by the numerical simulation. In the case of Cycle2, where the system is still not completely elastic, the presence of residual H-bonds contributes to a textural organization with acceptable barrier properties. The best compromise is then obtained for Cycle2 leading to a high cross-linking density and, therefore, acceptable mechanical properties, together with interesting *R*_p_ values, whereas extensive curing appears to favor undesired phenomena leading to the deterioration of the barrier properties of the 4EP-*p*PDA/oxide hybrid layer.

*R*_b_ values (Fig. 12b) appear to be directly related to the curing duration. The shorter the curing cycle, the higher the resistance of the bottom layer. This bottom part of the system appears again to be impacted by the curing of the resin, confirming the contribution of the organic part to the electrochemical properties of the bottom layer in favor of a full impregnation of the porous oxide by the resin.

Considering the values of the CPE *Q*_hl_ and *n*_hl_ parameters (Fig. 12c) associated with the hybrid layer, it can be observed that while Cycle4, Cycle3 and Cycle2 samples show almost identical values (about 5 × 10^−9^ F cm^−2^ s^*n*−1^ for *Q*_hl_ and about 0.95 for *n*_hl_), the system cured according to Cycle1 exhibits lower *Q*_hl_ values (about 2.5 × 10^−9^ F cm^−2^ s^*n*−1^) and slightly higher *n*_hl_ exponent values (about 0.98). This difference may be related to the average thickness of the hybrid layer. Indeed, the 190 °C temperature dwell of Cycle2, 3 and 4 starts before the gel point of the resin is reached during the 60 min during dwell at 170 °C, as the gel point is only reached after 70 min at 170 °C. Therefore, for these cycles, the benzoxazine still has a low viscosity at the end of the step at 170 °C, and increasing the temperature leads to the decrease of the viscosity of the resin, which can then deeper impregnate the porosity of the oxide layer. As the gel point is reached shortly after the start of the 190 °C temperature dwell, the thickness of the hybrid layer is fixed and does not undergo any further changes, explaining the identical CPE values obtained for Cycle4, Cycle3 and Cycle2 samples. However, for Cycle1 samples, the curing cycle is limited to 170 °C, resulting in a less deep impregnation of the oxide porous layer and, therefore, a slightly higher hybrid layer thickness and a lower CPE value. Indeed, the *n* values of the hybrid layers being close to 1, the CPE of the hybrid layer behaves like a capacitance, which decreases with thickness.

The comparison of the CPE *Q*_b_ and *n*_b_ parameters (Fig. 12d) is more complicated to interpret. Indeed, *n*_b_ values are different for all curing cycle, ranging from 0.5 up to 0.8. Such low values could be the consequence of a poor homogeneity of this part of the system. *Q*_b_ values appear to be lower for a shorter curing cycle. Such a trend could be related to the thickness and to the area of the bottom layer. Indeed, longer curing cycles may lead to the formation of more numerous and deeper resistive pathways through the hybrid layer down to the bottom part of the system, resulting in thinner bottom layers with a greater area and so an increase in *Q*_b_ values. However, though these conclusions should be made with great care, as the *n*_b_ exponent values are scattered between 0.5 and 0.8, such values are also representative of heterogeneous layers which can be described by a transmission line element in a porous structure.^29,39^

The observation of the EIS spectra and the fitting of the associated impedance data allow us to conclude that curing cycles with limited curing temperature and duration may offer promising perspectives to tune and optimize the achieved barrier properties of anodic layers sealed with the 4EP-*p*PDA resin.

## 4 Conclusions

4-Ethylphenol-*para*-phenylenediamine-based benzoxazine has been spin-coated and fully cured on anodized AA2024-T3 substrates. The curing process, by inducing the melting of the resin at high temperature, permits the complete impregnation of the porous oxide layer by the benzoxazine due to its low viscosity. It results in a hybrid organic–inorganic layer and, at the same time, a porous anodic surface texture, which is restored by the impregnation step, offering a favorable condition for anchoring of further applied top coats. This textured surface has been morphologically and chemically described by SEM-FEG observations and GDOES depth-profiling.

The necessity of well controlling the curing step for obtaining high and durable barrier properties has been assessed by performing EIS characterization of coated samples for up to one month of immersion in a saline electrolyte. Obtained barrier properties are very promising for coatings with such a low layer thickness (<4 μm). Despite the complexity of this system, the electrochemical properties have been fitted using a rather simple EEC, valid all over the immersion period. The barrier properties obtained by different limited curing cycles have been compared thanks to the fitting procedure. The curing process parameters appeared to have a strong impact on the achieved properties. Partial curing of the system is then a very promising way of respecting the thermal sensitivity of aeronautical alloys while optimizing the corrosion protection of aluminum alloys.

## Conflicts of interest

There are no conflicts of interest.

## Supplementary Material
